# Using critical race theory to reframe mentor training: theoretical considerations regarding the ecological systems of mentorship

**DOI:** 10.1007/s10734-020-00598-z

**Published:** 2020-08-25

**Authors:** Jose H. Vargas, Carrie L. Saetermoe, Gabriela Chavira

**Affiliations:** 1 California State University, Northridge, Northridge, CA, USA

**Keywords:** Critical race theory, Ecological systems/ecosystems, Mentorship, Pushout problem, Race/racism, Students of color

## Abstract

This article offers a theoretical and critical analysis of race-dysconscious mentorship involving students of color and white faculty. Inspired by *ecological systems theory*, *critical race theory*, and the NIH-funded program, *Building Infrastructure Leading to Diversity: Promoting Opportunities for Diversity in Education and Research*, our analysis considers the ecosystems that promote student pushout and hinder diversification of the scientific workforce, which call for “critical” alternatives to traditional research mentorship. We first examine the historical, social–political, institutional, interpersonal, and intrapsychic ecosystems of traditional mentor–protégé relationships. Two areas are reviewed: (a) “diversity” as it operates in universities and research laboratories and (b) the discursive properties of a dysconscious dialog that rationalizes modern racism. Next, we connect the five ecosystems of mentorship by integrating literature on critical history, white consciousness, the interpersonal context of mentoring, and mentor–protégé phenomenology. Our analysis demonstrates how the racialized lives of members involved in a mentoring relationship are situated within racist macro-level ecological systems wherein intrapsychic and interpersonal actions and discourses unfold. The development of race-consciousness and anti-racist faculty mentor training programs is also discussed.

The problem with individualism is …attributing to individuality the things produced only in the dialectic of interpersonal relations. Through this, individualism ends up reinforcing the existing structures, because it ignores the reality of social structures and reduces all structural problems to personal problems.

—Ignacio Martín-Baró 1994, p. 22

Structural racism limits higher education and every branch of the social and natural sciences. The Centers for Disease Control defines racism as “a system of structuring opportunity and assigning value based on phenotype (race) that unfairly disadvantages some individuals and communities; unfairly advantages other individuals and communities; [and] undermines…the full potential of…society” (Jones 2002, p. 10). Inside the academic-scientific community, a dearth of *diversity* (i.e., ideas from various identity groups; Thomas and Ely 1996) constrains imagination and critical problem-solving by essentializing, or making natural and immutable, culturally biased ecosystems that define merit in Western-individualist terms (Kosoko-Lasaki et al. 2006). Essentialized scientific practices quiet the voices of students of color who might develop innovative research programs that would make space for prosocial change in the sciences (Ponjuan 2011; Russell et al. 2015). These practices contribute to the *pushout problem*, whereby talented students of color prematurely quit research and self-deidentify from science (Holoien and Shelton 2012; Luna and Revilla 2013). We argue that race-dysconscious mentor training—devoid of Paulo Freire’s (1970) *concientización*, or social–political consciousness—culturally reproduces racism via the ecosystems of higher education, the academic-scientific community, and social life (see King 1991).

Paralleling *ecological systems theory* (Bronfenbrenner 1979), we deconstruct the interconnected and nested intrapsychic, interpersonal, institutional, social–political, and sociohistorical forces that affect mentor–protégé relationships between white faculty and students of color.^1^ Our analysis draws from *critical race theory* (CRT; see Crenshaw et al. 1995) and positions race/racism within these forces, so that they may be labeled and transformed in the service of “critical” mentorship models that counter white dominance in all its pervasive forms. For clarity, we refer to an ecosystems model as we introduce important concepts (Fig. 1). The model captures conceptual links key to our position. This position was inspired by a critical mentor training program operating out of the NIH-funded site, *Building Infrastructure Leading to Diversity: Promoting Opportunities for Diversity in Education and Research* (BUILD PODER), located at California State University, Northridge (CSUN; Saetermoe et al. 2017). While a case-study of BUILD PODER is beyond the scope of this article, its CRT philosophy is extremely nonconforming and requires that student and mentor training follow an anti-racist agenda. This offers a special canvas on which to draw contrasts of its critical approach to mentorship training against standard training practices. Both parts of this article begin with a macro-level account that transitions into a micro-level analysis, all the while drawing upon connections to CRT and BUILD PODER. In Part 1, we introduce the historical, social–political, and institutional factors that justify the CRT-based BUILD PODER program and then shift to an analysis of the racialized interpersonal and intrapsychic realities of power-unequal relationships. In Part 2, we reframe mentorship using CRT. By integrating research on critical history, the macro-level ecosystems of white consciousness, and the interpersonal context and phenomenology of mentorship, we reveal how the racialized lives of each party involved in this special bond are situated within racist ecosystems, wherein intrapsychic and interpersonal experiences, discourses, and actions unfold.

## An embedded relationship of unequal power: Situating the protege and the mentor in their ecosystems

**Fig. 1:**
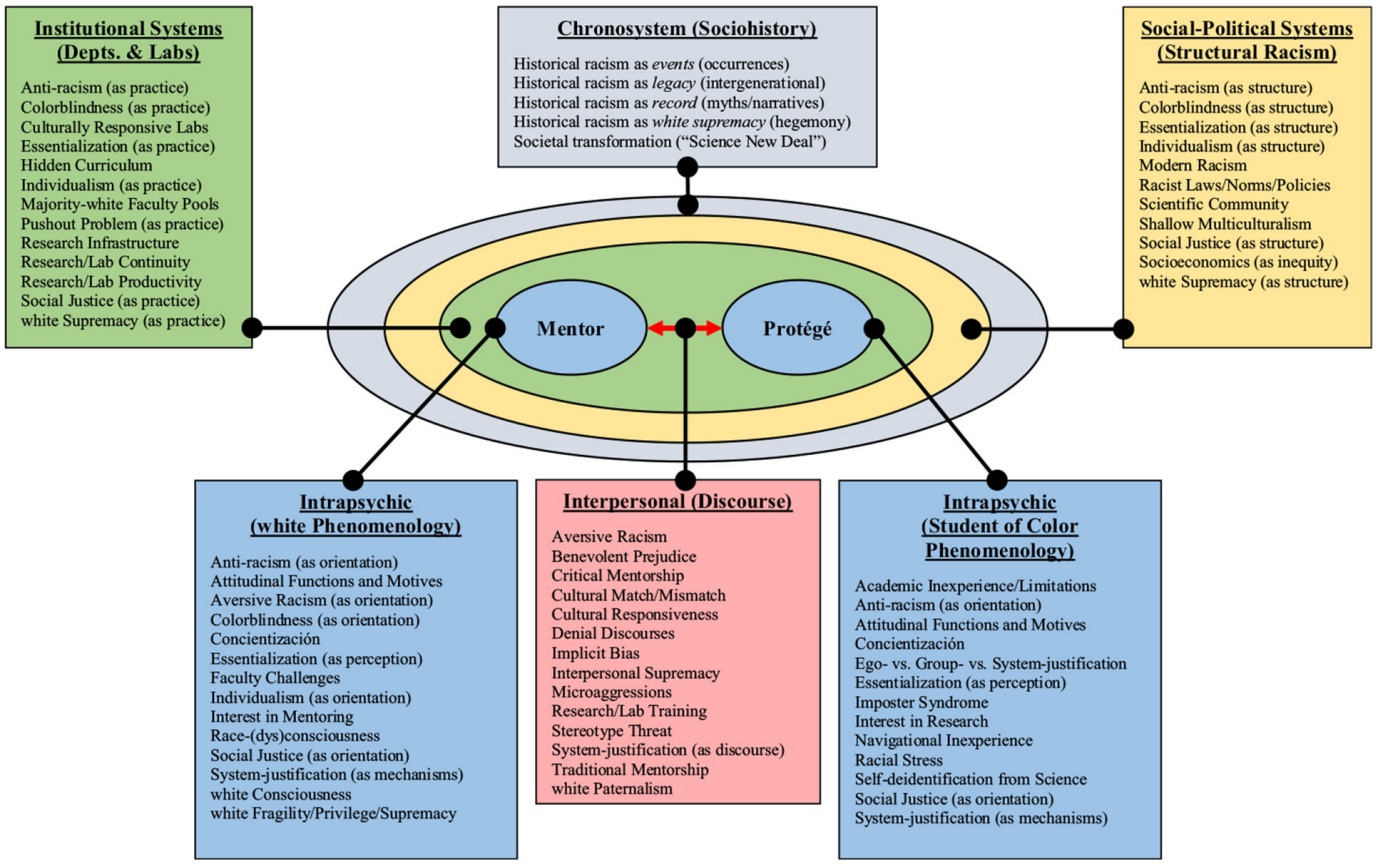
The intrapsychic, interpersonal, institutional, social–political, and chronosystemic contexts of mentorship. A Bronfenbrenner-type ecological systems model is used to depict the nested and recursive relationships between phenomenological experience, discourse, academic research institutions, structural racism, and sociohistory

Liberatory stances contend that elevating social consciousness requires deconstructing the *chronosystemic* and *social–political systems* of human experience (Freire 1970; Martín-Baró 1994). In the USA, social–political systems, or the laws, norms, policies, economics, and ideologies that encompass the structures of racism, are inseparable from historical legacies of white supremacy (Fig. 1). The pushout problem among students of color cannot be resolved if those seeking solutions neglect to situate Westernized mentorship within these powerful ecosystems. Accordingly, BUILD PODER uses CRT to confront three problems: (a) the racist sociohistorical and structural factors that provide a rationale for critical mentorship, (b) the institutional failures of the diversity promise, and (c) how system-justification, as an intrapsychic coping strategy, ties notions of fairness, individualism, and merit in ways that aid predictable interpersonal discourses of denial that obstruct the realization of systemwide ethnic/racial parity. We discuss these topics in turn.

### Critical race theory and BUILD PODER: The case for critical mentorship

Historical forces associated with white supremacy ripple through time into the present. Since the colonial period, both persons of color and whites residing in what is now the USA have suffered from the destructive effects of structural racism, albeit in distinct forms (Battalora 2013). Mentor training programs represent one area where CRT can be effective in diversifying white-dominated science workforces. The origins of CRT go back to the mid-1980s, when it began as a social movement in Harvard Law School’s course on race and law (Bell 1980; Crenshaw et al. 1995). Early proponents of CRT challenged the colorblind structure of traditional legal studies by embracing race-conscious critiques of US legal systems. Later proponents who found value in centering race, in both epistemology and praxis, would implement CRT tenets across other social scientific areas (Salter and Haugen 2017). Thus, CRT has a recent sociohistory of promoting race-conscious analyses of educational systems through critiques of their racist social–political facets (Yosso 2002).

Five CRT tenets promote the transformation of racist ecosystems: (a) the centrality of race/racism in human affairs, (b) a desire to label and dismantle oppressive social systems, (c) a commitment to social justice, (d) the pragmatic value of experiential knowledge, and (e) the need for interdisciplinary approaches and solutions (see Ladson-Billings 1998; Solórzano and Yosso 2002; Yosso 2002). The CRT tenets are fluid and interactive, offering both palpable and subtle ways of guiding theoretical development. Table 1 outlines the forms in which CRT contributed to our analysis. We argue that white faculty can be empowered to develop new research paradigms by incorporating their protégés’ scientific interests into research practice and by valuing protege experience (see Yosso 2005). With training, white mentors can learn to situate themselves in a structurally racist reality, to better understand their proteges, to become more productive scientists, and to give substance to the research experience as they nest the scientific community within the ecosystems of social life.

Created to diversify the biomedical workforce, CSUN BUILD *PODER* (the Spanish noun for “power”) is one of ten NIH-funded BUILD site training undergraduates in research. The CSUN site adopts an anti-racist agenda that considers the detrimental impact of historical and structural racism (Saetermoe et al. 2017). While some research programs focus exclusively on student enrichment (e.g., Plunkett et al. 2014), CSUN BUILD PODER supports students by transforming research culture through infrastructure, faculty research support, and professional training (see McGee et al. 2012). A central area of student support is the CRT-based mentor training program, which fashions bridges between culturally different faculty and students by unpackaging the role of racism in mentorship and research training (see Hassouneh-Phillips and Beckett 2003). The program advances CRT-informed strategies for respectful interactions by elevating critical consciousness and formulating intentional practices around race and its intersections with other social identities. It also encourages faculty to transform racist institutional practices—including unmet diversity promises—that lead to student pushout.^2^

### CRT tenet—Dismantlement: Deconstructing the diversity promise

Mentor–protégé relationships are nested within *institutional systems* that include practices, infrastructure, laboratory cultures, and personnel (Fig. 1). The CRT tenet of dismantlement compels us to note that in most US universities, students of color likely attend classes and work in laboratories headed by white faculty. Students of color are often trained at predominantly white institutions because of their sheer availability (Hrabowski III 2012). The National Center for Education Statistics reports that whites constitute over three-quarters (i.e., 76%) of full-time faculty in the USA; Asians/Pacific Islanders, African Americans/blacks, and Latina/o faculty comprise only 10%, 6%, and 5%, respectively, and less than 1% identify as American Indian/Alaska Native (DOEd, USA 2018). Historically racist university practices adversely impact student belongingness and scholastic outcomes, chiefly for students of color (Kosoko-Lasaki et al. 2006; Ponjuan 2011). As the US populace becomes more diverse, and as universities struggle to diversify faculty pools, culturally mismatched mentor–protégé relationships are likely to remain the norm for future students of color (see Weinberg 2008).

To transform racist institutions, the BUILD PODER mentor training program exposes faculty to the institutional facets of modern racism. Faculty learn that the power to institutionalize and reproduce inequity in higher education is made possible through a collective activation of social–political factors and corresponding institutions that include, inter alia: (a) “race-neutral” or colorblind policies (Aguirre Jr. 2010; Solórzano and Yosso 2002); (b) the ascription of individual- and deficit-based attributes to students of color (Viesca et al. 2013); (c) the use of rigid definitions of “merit,” which fail to integrate the strengths of students of color (Ledesma and Calderón 2015); and (d) the misuse of multiculturalism, which “otherizes” non-whites through superficial celebrations of cultural difference that neglect opportunities to critique the structural contributors to racism (Almeida et al. 2011). The CRT-based program also exposes faculty to the social–political properties of racism by linking to mentorship the deleterious effects that come with unequal power, the elevation of whiteness, the erasure of non-dominant social identities, and racist policies. In addition, we argue that institutional practices that promise (but work against) diversity do not arise out of a vacuum. Racist practices are thrusted by system-justifying attitudes and denial discourses that reify the ecosystems impacting mentor–protégé relationships. Thus, the reproduction of the pushout problem within the sciences cannot be fully recognized and addressed without due consideration of the actors who, via interaction and discourse, collectively contribute to the continuity of racist ecosystems.

### CRT tenet—Interdisciplinary work: System-justification, individualism, denial discourses, and the cultural reproduction of racism

Mentor–protégé relationships are *interpersonal systems* (Fig. 1). The BUILD PODER program addresses this ecosystem by exposing faculty to the science of implicit bias, microaggressions, and stereotype threat and by connecting interpersonal dynamics to structurally racist ecosystems. This knowledge, while valuable, requires further expansion. We contend that the program can improve by highlighting the specific discursive tools white mentors need to appreciate the experiences of students of color. To label and deconstruct this discourse-driven dyadic relationship in terms of its institutional, social–political, and chronosystemic contexts, the CRT tenet of interdisciplinary work is paramount. We turn to three social psychology areas: *traditional*, *cultural*, and *discursive*.

#### System-justification and cultural reproduction

The pushout problem is a product of a recursive macro–micro-systemic process, though faculty are rarely aware of these dialectics. That is, pushout results from a series of race-dysconscious interpersonal encounters that reinforce the status quo. Traditional social psychology reveals that legitimizing beliefs, stereotypes, and system-serving ideologies uphold preexisting hierarchical arrangements and discriminatory social–political systems (Jost and Burgess 2000; Jost and Hunyady 2002). *System-justification theory* holds that oppressive systems are reproduced when people desire to defend idiosyncratic stakes in the status quo, even if defensive action fails to serve group- or self-interest (Jost and Banaji 1994; Jost et al. 2004). Many stakeholders in academia share the erroneous idea that the status quo functions fairly and justly. A fallacy exists that if a racist system has the mark of functionality, then it must follow that the system is legitimate; otherwise, “reasonable” (or nonracist) individuals would not permit its existence in the first place. This tautologous mindset carries implications for mentor training. It rationalizes the presumption that traditional mentorship requires no modification, despite the ubiquity of racism. System-serving presumptions reduce the structural down to the personal and neutralize the racialized institutional and social–political ecosystems of everyday experience. In sum, colorblind ideologies and individualism work in tandem to perpetuate racist and destructive social orders that most white faculty are dysconscious toward.

#### Colorblindness and individualism

A common system-justifying ideology is *colorblindness*. Colorblind ideologies reproduce oppressive systems by decentering—or eliminating—the concepts of race and racism from all discourse and redirecting dialogic energy toward race-neutral conversations about competition, equality, and individualism (Bonilla-Silva 2003). Colorblind attitudes drive student pushout by promoting a form of assimilationism that silences diverse voices, resists cultural responsiveness, and is hostile to non-individualist and non-Western cultures (see Augoustinos and Every 2007, 2010; Augoustinos et al. 2005; Giroux 2003). Variations of the “I-don’t-see-color” refrain exemplify discursive ploys applied, intentionally and unintentionally, in the service of racist meritocracies (Wingfield 2015). Yet, cultural social psychology shows that in the USA, conceptualizations of independent selfhood are inseparable from attitudes about race, natural hierarchies, self-determination, personality, and capitalism (Bellah et al. 1986). Meta-analytic research shows that European Americans endorse *vertical individualist* values to a greater degree than African and Latina/o Americans, meaning that the white strand of the independent self-construal is tightly integrated with beliefs about the rightness of social dominance and competition (Vargas and Kemmelmeier 2012). Vertical individualism manifests itself in classrooms and laboratories, leaving students who approach problems cooperatively experiencing alienation and, ultimately, *deidentification* from the sciences (see Kagan 1992). This is problematic because Vargas and Kemmelmeier also show that among Latina/o Americans, a competitive orientation conflicts with expressions of solidarity with valued equal-status peers; also, for Asian Americans, personal autonomy aligns with egalitarian-collectivist values and, for African Americans, self-construal is less dependent on dominance and more informed by personal uniqueness. Colorblind ideologies ignore these cultural nuances and provide content for discourses that deny racism and that uncritically rationalize student pushout.

#### Discourses of denial, attitudinal functions, and system-justifying mechanisms

The denial of racial constructs acts as a barrier to authentic communication about racism inside academia (Augoustinos and Every 2010). According to discursive social psychological research, race-dysconscious attitudes fuel system-serving *discourses of denial*, or rejection narratives about racism, sexism, classism, and other modes of social injustice (see Augoustinos and Every 2007; Solomon et al. 2005). Race-dysconscious discourses, nurtured through institutional and social–political ecosystems of white prioritization, fallaciously reduce structural racism to individual choice and personal moral responsibility (Augoustinos et al. 2005; Giroux 2003).

System-justifications, expressed as denial discourses, reproduce student pushout through various mechanisms. These mechanisms shut down critical analysis about structural racism, ameliorate psychological distress, and restore legitimacy in the status quo. Table 2 provides a non-exhaustive typology of 10 commonplace system-justifying mechanisms and includes descriptions of their corresponding discursive features (see Gaertner and Dovidio 1986; Henry and Sears 2008; Lewis-Charp 2003; Malik 2000). Traditional social psychology argues that these mechanisms contain attitudinal components. We classify the 10 mechanisms by *attitudinal function*, or goal served by an attitude (Kruglanski and Stroebe 2005). Attitudes accomplish personal and social aims. Attitudes allow people to feel positive self-regard (*ego-defensive function*), simplify social cues (*knowledge-management function*), navigate social relationships (*social-adjustive function*), achieve rewards and avoid costs (*utilitarian function*), and convey self-relevant information (*value-expressive function*; see Fabrigar et al. 2005).

Attitudinal functions reveal motives associated with system-justifying discourses that disrupt culturally mismatched mentor–protégé relationships. Theoretically, such motives adversely impact these relationships in at least five ways. First, white mentors may hold self-validating ego-defensive attitudes that stigmatize the lives and customs of students of color (*pathologization*) or weaponize oppressed statuses (*victimization*). Second, knowledge-management attitudes are heuristic and may compel mentors to cognitively simplify complex culturally mismatched interactions by dismissing legacies of oppressive social–political systems (*ahistoricization*) or by denying the reality of modern racism (*denialism*). Third, through social-adjustive attitudes, white mentors may welcome racially dysconscious relationships with students of color, especially if mentors possess parallel beliefs about the “pragmatic value” of conformity (*assimilationism*) or race-neutrality (*colorblindness*) in sustaining social harmony. Fourth, utilitarian attitudes are instrumental and may provide white mentors with unsuitable interpersonal tools that create distance with students of color (*avoidance/aversive racism*) or explicitly rationalize inequity in research education (*meritocracy/symbolic racism*). Finally, value-expressive attitudes may grant white mentors the privilege to live by cherished standards perceived to be normative in US culture, like the elevation of the dispositional/person above the situational/system (*individualization*) or the defense of equality in lieu of equity (*equality/sameness*).

System-justifying mechanisms akin to those listed in Table 2 shape the lived experiences of both proteges and mentors. Through discourse, they guide the social construction of variant realities (across *separate* individuals) and allow for the simultaneous existence of those realities (across *shared* ecosystems). In line with this reasoning, system-justification theory informs how mentors and protégés differentially construct their phenomenologies. The theory predicts that among white faculty, the social, economic, and political benefits of structural racism are likely to generate a sense of appreciation in, identification with, and colorblind fidelity to the status quo; among students of color, the same ecosystems are likely to generate a sense of intrapsychic unease—a defensive state involving conflicts between desires for self-consistency, or *ego-justification*; desires for belonging through valued (and valuable) groups, or *group-justification*; and an abstract need to obligate oneself to a legitimate *superordinate* social system, or system-justification (see Jost and Banaji 1994; Jost et al. 2004). The revelation of system-justifying mechanisms unmasks racially dysconscious aspects of phenomenology that limit the formation of authentic and long-term mentor–protégé relationships and that likely explain instances of pushout.

## The higher-order ecosystems of whiteness and mentor–protégé phenomenologies

The BUILD PODER mentor training program advances the philosophy that value exists in confronting the subjective and racialized experiences of both the oppressor and the oppressed. Figure 1 illustrates that every mentor–protégé relationship is a unique dyadic bond between two phenomenologically distinct actors who are nested within higher-order recursive ecosystems. Social actors bring with them their own racialized histories, cultural worldviews, and subjective experiences into all interpersonal situations (DiAngelo 2018). These experiences make up the *intrapsychic system*. Intrapsychic experiences are differentially affected by higher-order ecosystems and system-justifying norms that fortify denial discourses about race and other topics construed as controversial or taboo. To the detriment of science, most white faculty do not receive the requisite training to understand and appreciate the racialized structural and psychosocial forces that impact their own behavior or how their behavior affects students of color (see Wallace and Brand 2012). As such, student pushout becomes normative. In this section, we address these problems in mentor training by appealing to the CRT tenets of race/racism centrality, experiential knowledge, and social justice. Specifically, we analyze the chronosystemic and social–political contexts of whiteness. Next, our analysis narrows in on the interpersonal and intrapsychic contexts of mentorship. From this, we reframe traditional mentorship and advocate for critical models that actively disrupt the recursive ecological systems of modern racism.

### CRT tenet—Race/racism centrality: Contextualizing “whiteness” as property and the myth of individualism

The BUILD PODER program argues that phenomenology cannot be disentangled from its chronosystem, or the sociohistorical backdrop of lived experience (Fig. 1). A judicious discussion on the role of whiteness in the pushout problem requires applying the CRT tenet of race/racism centrality through a fusion of critical history. Historically, whiteness as a group-level attribute has always bestowed tangible benefits (Harris 1993). Ironically, the rhetoric around self-reliance and merit within academic institutions, as explanation for the successes and failures of students of color, grew out of collectivist ecosystems (e.g., multi-family communities, churches, schools, and legislatures) dominated by Anglo-Saxon Protestants (Battalora 2013; Shain 1994). In higher education, the economic, political, and social privileges bequeathed to white faculty are mainly invisible and taken for granted due to years of ahistoricism, denialism, and other system-justifying discourses.

Although all faculty possess intangible educational privileges (e.g., status), white people possess additional privileges which are more *transactional* in nature (e.g., policy control) and which serve as limitless “property” that—like land or money—is used ubiquitously across many social interactions. Harris (1993) advanced this thesis, labeling it *whiteness as property*. Three pillars are central to this thesis. First, from an economics framework, a series of colonial uprisings during the late seventeenth century (e.g., Bacon’s Rebellion; Battalora 2013; Kendi 2016) stimulated a need to fracture disenfranchised black-white alliances, coalesce poor and rich “whites,” and consolidate minority (aristocratic) rule. Second, regarding ideology, the need to institutionalize and enforce white supremacy led to the construction of political discourses and legal lexicons pertaining to whiteness and race. Finally, at the social level, and given the economic, legal, and political benefits of white supremacy, the *popular* construct of whiteness transformed into a social commodity that would garner material returns (Wise 2017). Thus, whiteness—as metaphysical property restricted to white people within a *racial caste system*—impacts actual wealth, education, health, and other social life domains due to policies, laws, and practices that remain hidden through layers of individualism, multiculturalism, and a colorblind credo. Its interpersonal and experiential consequences are disastrous.

### CRT tenet—Experiential knowledge: The interpersonal context of traditional mentorship

The BUILD PODER program holds that the interpersonal dimensions of culturally mismatched mentorship are dynamic and conflictive (Fig. 1). To disrupt race-dysconsciousness among culturally mismatched mentors and protégés, the program finds it necessary to tap into the experiences that occur in these relationships. As such, our ecosystems framework leans on the CRT tenet of experiential knowledge, which holds that practical value exists in deconstructing racialized “relational” experience. Indeed, mentors view themselves as open to having holistic relationships with their protégés and are committed to supporting diversity (Ahmad et al. 2019). Yet, racist social–political systems make racism invisible to advantaged people, and even well-intentioned mentors cannot always distinguish intent from impact. Instructors who are white and concerned about appearing racist may give limited feedback to students of color (Harber et al. 2010) or may experience impatience with, ignore, or hold low expectations for students of color (Suarez-Balcazar et al. 2003). For instance, faculty tend to encourage white male students, compared with students of color and female students, to pursue doctorates (Kim and Sax 2009). Moreover, some faculty are aware of their inexperience with racism and avoid discussions about race (Sue et al. 2009).

In this fraught interpersonal context, students of color and white mentors hold dissimilar visions of the research experience and the mentor–protégé relationship (Dodson et al. 2009). Studies have found that faculty misattribute student researcher turnover to lack of interest while only 10% of students report this motive (Seymour and Hewitt 1997). Arguably, without the long-term investment needed to generate interpersonal trust around racial issues, instances that foster system-justification can reinforce an environment of miscommunication, misattribution, mutual silence, and pushout. Thus, whiteness perpetuates a style of *interpersonal supremacy* that goes unnoticed by white faculty; in contrast, relational barriers are salient to students of color, who are burdened with the costs of multiculturalism, white sensibilities, and aversive racism.

#### The problem with multiculturalism

The BUILD PODER mentor training program teaches that pushout is partly a product of multiculturalism, which masks structural racism. It hides intergenerational legacies of white hegemony—over curricula, research paradigms, and definitions of merit—and leaves racism unchecked (Hassouneh-Phillips and Beckett 2003). We theorize that misuses of multiculturalism adversely influence white phenomenology and white mentors’ attitudes toward students of color (Fig. 1). Multicultural practices that demand a colorblind and assimilationist stance prevent progress in anti-racism movements by otherizing students of color and by celebrating superficial differences that make whites feel culturally competent as they fail to appreciate the seriousness of recurrent racism (Almeida et al. 2011). Racist ecosystems, in conjunction with mentors’ personal satisfaction in their own cultural “competence,” allow for unexamined biases that individualize or pathologize the assets of students of color (McCoy et al. 2015; Yosso 2005), normalize low expectations, and reinforce habitual and subtle acts of interpersonal supremacy (e.g., microaggressions) that may better explain the pushout problem (Holoien and Shelton 2012; Luna and Revilla 2013).

#### The problem with white sensibilities

BUILD PODER also acknowledges that anti-racist efforts are often met with resistance. When confronted with the history and social–political ecosystems that favor whiteness, an intrapsychic equilibrium is disrupted that leads whites to experience predictable emotional states, such as feeling defensive, angry, guilty, misunderstood, or censored (see Bonilla-Silva 2003). DiAngelo (2011) uses the term *white fragility* to describe this intolerance for racial stress. It is engendered by media, education, law, and other aspects of social life that normalize white Christian heteronormative lifestyles and that overlook de jure and de facto physical- or social-based ethnic/racial segregation and discrimination (see Tatum 1997). In mixed-ethnicity/race interactions, white fragility protects white hegemony through a collective process that equates “structural racism” with malicious acts performed by morally deficient people, and whose definition becomes inherently non-structural, purely individualistic, and disassociated from the history of white supremacy (DiAngelo 2018). As such, white fragility constrains critical analysis, suppresses race-consciousness, and actively reproduces the pushout problem.

#### The problem of aversive racism

Uncritical cultural competence education and white fragility may explain certain manifestations of aversive racism (see Young and Davis-Russell 2002). Gaertner and Dovidio (1986) characterize aversive racism as an attitudinal ambivalence toward, and behavioral avoidance of, ethnic/racial outgroups. We conceptualize aversive racism in both intrapsychic and interpersonal terms (Fig. 1). In culturally mismatched mentor–protégé relationships, whites may openly deny racially motivated behavior while concurrently expressing trepidation toward students of color (see Prunuske et al. 2013). Denialism, along with the neurocognitive demands required to actively monitor the leakage of unconscious bias, may motivate white mentors to avoid difficult discussions around racism (McCoy et al. 2015; Sue et al. 2009). Similarly, white mentors may alter their behaviors to appear nonracist and hold low standards for students of color, thus displaying a type of white paternalism or *benevolent prejudice* that reifies inequity by failing to fairly challenge students of color (see Jackman 1994; Whitley Jr. and Kite 2010).

### CRT tenet—Experiential knowledge, revisited: The phenomenologies of protégés of color and “white” mentors

The BUILD PODER program also acknowledges the intrapsychic dimensions of culturally mismatched mentor–protégé relationships (Fig. 1). The success of the anti-racist program depends on juxtapositions of both protégé and mentor phenomenology, or the “personal” experiences of one member in the absence of the other. Therefore, our ecosystems framework once again leans on the CRT tenet of experiential knowledge. We briefly discuss these two phenomenologies.

#### On protégés of color

To transcend the interpersonal barriers associated with cultural mismatch, the BUILD PODER program encourages faculty to confront the phenomenologies of their proteges. The intrapsychic systems of students of color are dynamic and involve conflicts between ego-, group-, and system-justification, self-doubts about scientific efficacy (“the imposter syndrome”), and racial stress (Fig. 1). Students of color in science, on top of learning rigorous curricula, face added challenges. Students of color often justify a racist status quo in order to gain access to higher education, deal with few mentors they can trust, fight off efforts toward marginalization, and learn culturally irrelevant curricular material (Ortiz and Jani 2010). Moreover, structural factors, like complex application forms, multiple bureaucracies, and assimilationist policies lay the groundwork for individualistic, competitive, and hidden curricula that can be daunting to students of color (Byars-Winston et al. 2018). These racist structures take a psychological toll on students who hold great promise but whose experiences have been unwelcomed, as institutions have yet to recognize and accommodate the lived realities of talented persons of color (NIH 2012).

#### On “white” faculty mentors

Identifying ways to address the pushout problem via rapport and trust building requires an appreciation of white phenomenology (Fig. 1). Mentorship can be viewed as a hurdle by novice faculty who must reconcile time pressures, new experiences, research and service demands, teaching loads, and other realities (Jacobs and Winslow 2004). Furthermore, white fragility and system-justifying discourses that serve ego-defensive, knowledge-management, social-adjustive, utilitarian, and value-expressive attitudinal functions provide white mentors with race-dysconscious interpersonal tools that redirect much-needed critical-racial dialog, ameliorate racial distress, and restore systemwide legitimacy (Table 2). Despite these challenges, faculty find mentorship worthwhile and central to their identity as academics (Potter et al. 2009). They recognize that mentorship makes a meaningful difference in the lives of students of color (Jackson et al. 2003). Hence, we argue that anti-racist laboratory cultures that are responsive to the needs and experiences of students of color, and that are championed by race-conscious white mentors, will create space for social cohesion and group morale, which will positively impact research continuity and productivity (see Carpi et al. 2017; Sabat et al. 2017). We suspect that race-conscious research cultures are predicated on the willingness of white mentors to overcome intrapsychic hurdles that interrupt authentic mentor–protégé communication.

### CRT tenet—Social justice: Cultural match/mismatch and reframing mentorship with “white” allyship

In accord with the CRT tenet of *social justice*, we advocate for the institutionalization of anti-racist and critical mentor training as a way to dismantle and transform structurally racist, colorblind, and predominantly white academic ecosystems. Given that most students of color attend universities with majority-white faculty pools, cultural mismatch between mentors and protégés of color is the norm rather than the exception (Hrabowski III 2012). This is unfortunate. Cultural match can allay status differences for students of color (Santos and Reigadas 2004). An ethnically or racially similar mentor can serve as a role model to remind protégés of color of what is possible (Almeida et al. 2011; Hendrix 1998). Also, faculty of color can recognize and respond to discrimination and racial stress (see McCoy et al. 2015). Some research even indicates that students of color are more likely to receive assistance from faculty of color than from white faculty (Blake-Beard et al. 2011).

The findings above favor permanent actions that diversify faculty pools and give faculty of color institutional support to role-model the requisite skills students of color must learn to succeed in research and in their careers (Quaye et al. 2015). In the interim, white mentors must leverage their positions of white advantage in order to fill this educational void (see Gibbons 1993). Social justice demands it! Obstacles related to banal white privilege and racism remain ever-present and continue to impede parity in the science workforce. Alliances between anti-racist white mentors and students of color are needed to change the status quo for the better. Mentors who are white and opt to incorporate critically informed social justice themes in scientific research can personify the CRT tenet that research must make positive change. Through concientización and nurturant bonds, white mentors can motivate students of color to become researchers/scientists and to apply science in ways that improve the entirety of society (Saetermoe et al. 2017).

## Discussion

Diversification of the sciences depends on welcoming culturally informed and innovative voices. Presently, laboratories and professoriates do not reflect the general population or the student body in terms of ethnicity and race, including their intersections with other pertinent social identities. Anti-racist efforts in the service of diversification must include the construction of bridges between white mentor allies and students of color, whose experiences differ remarkedly. As the USA moves toward greater diversity, a need will grow to institutionalize critical mentorship approaches—akin to the BUILD PODER program—that properly serve students of color, who are the future of science and who are best equipped to operationalize and solve the needs of their valued (and valuable) communities. Much of BUILD PODER’s programmatic success derives from its implementation of the CRT tenets of race/racism centrality, dismantlement, social justice, experiential knowledge, and interdisciplinary work. These tenets can guide other universities in their quests to create anti-racist practices and institutions via faculty mentorship training.

As Fig. 1 illustrates, the intergenerational, structural, institutional, discursive, and phenomenological aspects of social life cannot be overstated. Founded upon bad-faith alliances forged between poor and wealthy whites during the colonial period, system-justifying ideologies—in modern times—mask recurrent white supremacy and preclude the self-realization that the burden of achieving racial justice falls upon white people (see hooks 1995). Mentors who are white and who avoid critical discussions about racism maintain hostile social climates for promising students of color (see DiAngelo 2018). As BUILD PODER and CRT exemplify, white mentors can be made aware of the nested ecosystems that impact their research and learn to acknowledge diverse perspectives holistically and without judgment (see Ahmad et al. 2019). Anti-racist/critical mentors can awaken within protégés a sense that science can resolve disparities that exist in disadvantaged communities throughout the world. By reaching into the phenomenologies of protégés, and by inviting their ideas into the scientific community, mentors can transform the role of research beyond a system-justifying practice that has yielded too little with respect to social justice. Anti-racist science practices, informed by culturally relevant knowledge and CRT, can retain greater numbers of novice scientists who live out the implications of their work and efforts (see Sabat et al. 2017).

### Recommendations and future directions

Social–political consciousness is a process, not a competency. Self-education and interdisciplinary perspectives can initiate this lifelong venture. In line with CRT, it is essential to learn about the racialized history of the USA, not as told through the mythologies of the oppressor, but as told through the voices of the oppressed and through critical social science and scholarship. An abundance of CRT-based literature from the social sciences and humanities, much of which informed this article, is readily available. Sources like Acuña’s (2019) *Occupied America*, Battalora’s (2013) *Birth of a White Nation*, Bonilla-Silva’s (2003) *Racism without Racists*, DiAngelo’s (2018) *White Fragility*, Ignatiev’s (1995) *How the Irish Became White*, Kendi’s (2016) *Stamped from the Beginning*, and Shain’s (1994) *The Myth of American Individualism*, among others, can serve as foundational knowledge for white mentors who are unfamiliar with race-dysconsciousness or who aspire to leverage the anti-racist power of concientizacion throughout their careers and in their personal lives. Once white faculty realize that race is a social construct conceived with the sole intent to favor *wealthy whites* over all others, they can begin to recognize how the assimilationist paradigm of the scientific enterprise wastes diverse talent and curtails much-needed innovation.

The social returns of CSUN BUILD PODER, especially its CRT-based faculty mentor training model, demand further investigation and multi-university implementation. Anti-racist faculty mentors can transform science by retaining a new generation of scientists of color who are motivated to make change for the betterment of humankind. Knowing how racist legacies influence research priorities may move the scientific community toward transforming its meritorious view of academic excellence. Allowance for paradigms that integrate student of color experiences gives space to a critical scientific philosophy that is cognizant of the ecosystems of inequity and how people and societies are compromised by racism and race-dysconsciousness. To transcend these problems of living, mentors and protégés must negotiate a new interpersonal contract and work as partners in championing a “Science New Deal” that moves beyond narrow and prescribed scientific principles and practices to critical principles and practices that actively counter destructive and racist social orders (see Maxwell 2007).

## Conclusion

This article offered a theoretical and critical analysis of race-dysconscious mentor training. Drawing from the CRT tenets of race/racism centrality, dismantlement, social justice, experiential knowledge, and interdisciplinary work, our interdisciplinary race-centered analysis deconstructed the ecosystemic contexts that hinder diversification of the science workforce and that justify the institutionalization of critical alternatives to traditional mentorship training for white faculty who mentor students of color. The racialized experiences of protégés and mentors must be situated within the racist ecosystems where interpersonal interactions and discourses occur. A major contribution of CSUN BUILD PODER is its premise that diversifying the scientific community is vital if social justice is to be achieved. In action, students of color will benefit from the concientización of white faculty who are conscious of the historically entrenched and structurally reinforced racist policies and practices that create impediments for budding scientists. By eschewing system-justifying discourses and conceding that educational opportunities are not fairly distributed across communities, racist ecosystems can be dismantled and transformed to mete out equity for all students. As academic institutions train diverse student scholars, they must also nurture critical faculty mentor teams to ensure the success of students of color, whose indispensable knowledge about the priorities, values, and customs of their families and neighborhoods could improve scientific reasoning, questioning, methodology, analysis, and interpretation in ways previously dismissed, unimaginable, or feared.

## Figures and Tables

**Table 1:** CRT Tenets, their Applications, and their Placement in Figure 1

CRT Tenet	Application	Ecological Systems Component	Sample Source
Dismantlement:	Deconstructing the diversity promise	Institutional Systems	Aguirre Jr. (2010): Hrabowski III (2012):Solórzano Yosso (2002)
	Traditional mentorship	Institutional Systems	Prunuske et al. (2013)
Experiential Knowledge:	Aversive racism	Interpersonal Systems	Gaertner & Dovidio (1986)
	Benevolent prejudice (white paternalism)	Interpersonal Systems	Whitley Jr. & Kite (2010)
	Student of color phenomenology	Intrapsychic Systems	Ortiz & Jam (2010)
	white phenomenology (race-dysconsciousness and white fragility)	Intrapsychic Systems	Bonilla-Silva (2003): DiAngelo (2011, 2018); King 1991
Interdisciplinary Work:	Cultural social psychology (vertical individualism)	Interpersonal Systems	Shain 1994; Vargas & Kemmelmeier (2012)
	Discursive social psychology (discourses of denial)	Interpersonal Systems	Augoustinos & Every (2007, 2010)
	Education research	Social-Political Systems	Ladson-Billings (1998); Yosso (2002, 2005)
	History (whiteness as property)	Clironosystems	Battalora (2013): Harris (1993)
	Traditional social psychology (attitude function theory and system-justification theory)	Interpersonal Systems	Jost & Banaji (1994); Kruglanski & Stroebe (2005)
Race/Racism Centrality:	Multiculturalism (colorblindness)	Social-Political Systems	Almeida et al. (2011); Bonilla-Silva (2003)
	Pushout problem and racism	Institutional Systems	Holoien & Shelton (2012); Luna & Revilla (2013)
	Structural racism and white hegemony/supremacy	Social-Political Systems	Battalora (2013); Kendi (2016)
Social Justice:	Anti-racist science communities	Institutional Systems	Ahmad et al. (2019); Saetermoe et al. (2017)
	Concientizacion (social-political consciousness)	Intrapsychic Systems	Freire (1970): Martín-Baró (1994)
	Reframing traditional mentorship (anti-racist mentors)	Interpersonal Systems	Saetermoe et al. (2017); Yosso (2002, 2005)
	Societal transformation via science (“Science New Deal”)	Chronosystems	Maxwell (2007)

*Note*. The five tenets of critical race theory (CRT): desire to label and dismantle oppressive social systems; pragmatic value of experiential knowledge; interdisciplinary approaches and solutions; centrality of race and racism in human affairs; and commitment to social justice

**Table 2.** Typology of System-justifying Mechanisms and their Corresponding Attitudinal and Discursive Properties

Attitudinal Function	Attitudinal Motive	System-justifying Mechanism	Denial Discourse Theme	Sample Dialogue
Ego-defensive:	Maintenance of positive self-regard and self-esteem	Pathologization:	Stigmatization and abnormalization of practices that, and people who, do not conform to the status quo	Minorities are disadvantaged because of their unwillingness to work hard enough. Unlike us, minorities don’t know how to raise successful children.
		Victimization:	Weaponization of the victim status	Minorities should stop complaining and just pick themselves up. People who are white are victims of reverse-racism.
Knowledge-management:	Cognitive/mental simplification of social cues and interactions	Ahistoricization:	Belief in the immutability, universality, naturalness, invariability, or eternality of the status quo	I’m not responsible for the racist acts that happened centuries ago. Racism is a thing of the past.
		Denialism:	Bad- and good-faith skepticism regarding the actuality of contemporary structural racism	Racial discrimination is no longer a serious problem for minorities. Modern racism does not really exist.
Social-adjustive:	Navigation of valued, necessary, or important social interactions and interpersonal relationships	Assimilationism:	Justification of exclusion and marginalization	Minorities would do better in life if they just assimilate. Minorities should learn to speak English.
		Colorblindness:	Fidelity to context-independent ethnic- and racial-neutralism	There’s only one race; the human race. I don’t care if someone is black, white, brown, yellow, red, purple, green, or polka-dotted.
Utilitarian:	Achievement of social rewards and avoidance of social costs	Avoidance/Aversive Racism:	Avoidance of self-reflection or social interactions with underprivileged people and other definable outgroups	People tend to group with their own people. You can’t force people together.
		Meritocracy/Symbolic Racism:	Belief in distributing resources in Western terms of achievement, talent, (“general”) intelligence, worth, and credentials	It’s not about race, it’s about ability and talent. Minorities have gained advantages that are undeserved, like affirmative action programs.
Value-expressive:	Conveyance of self-relevant beliefs, values, and personal information	Equality/Sameness:	Preference for equality in lieu of social justice or equity (fairness)	I don’t believe in special treatment for any group. The continuing demands of minorities are unwarranted.
		Individualization:	Proclivity toward disposition-based attributional reasoning in lieu of situation-based attributional reasoning	The system is not to blame; people are responsible for their own actions. We should all proudly identify ourselves as independent Americans.

*Note*. System-justifying mechanisms function in ways that are additive, interactive, and context-dependent. The 10 mechanisms represent commonplace system-justifying processes, which redirect uncomfortable dialogue, ameliorate emotional distress, and restore legitimacy in the status quo. Color has been added for the purposes of reading clarity

## Footnotes

1 We use *protégé* in lieu of *mentee*. The latter connotes a transactional affiliation, whereby students are treated as receptacles of another person’s knowledge. The former denotes a mutual bond, whereby students are treated as nascent professionals (see Graves II 2013). Also, we decline to assign proper noun status to race-dysconscious nomenclature like “white” or “black.” These labels essentialize race and reproduce structural racism (cf. APA 2020).

2 Following the murder of George Floyd and the reinvigorated Black Lives Matter movement, BUILD PODER faculty and allies formed an “End the Silence” group. Motivated by the CRT tenet of social justice, the purpose of this anti-racist group is to help CSUN personnel develop race-consciousness through open and critical dialog, so that they may transform race-dysconscious practices in their own workspaces and personal lives.

## References

[R1] Acuña RF (2019). Occupied America: a history of Chicanos (9th ed.). Boston: Pearson.

[R2] Aguirre A Jr. (2010). Diversity as interest-convergence in academia: a critical race theory story. Social Identities, 16, 763–774. 10.1080/13504630.2010.524782.

[R3] Ahmad AS, Sabat I, Trump-Steele R, & King E (2019). Evidence-based strategies for improving diversity and inclusion in undergraduate research labs. Frontiers in Psychology, 10, 1–6. 10.3389/fpsyg.2019.01305. 31316412 PMC6611382

[R4] Almeida R, Hernandez-Wolfe P, & Tubbs C (2011). Cultural equity: bridging the complexity of social identities with therapeutic practices. International Journal of Narrative Therapy & Community Work, 3, 43–56.

[R5] [APA] American Psychological Association. (2020). Publication manual of the American Psychological Association (7th ed.). 10.1037/0000165-000.

[R6] Augoustinos M, & Every D (2007). The language of “race” and prejudice: a discourse of denial, reason, and liberal-practical politics. Journal of Language and Social Psychology, 26, 123–141. 10.1177/0261927X07300075.

[R7] Augoustinos M, & Every D (2010). Accusations and denials of racism: managing moral accountability in public discourse. Discourse & Society, 21, 251–256. 10.1177/0957926509360650.

[R8] Augoustinos M, Tuffin K, & Every D (2005). New racism, meritocracy, and individualism: constraining affirmative action in education. Discourse & Society, 16, 315–340. 10.1177/0957926505051168.

[R9] Battalora J (2013). Birth of a white nation: the invention of white people and its relevance today. Houston: Strategic Book Publishing and Rights Company.

[R10] Bell DA (Ed.). (1980). Shades of Brown: new perspectives on school desegregation. New York: Teachers College Press.

[R11] Bellah RN, Madsen R, Sullivan WM, Swidler A, & Tipton SM (1986). Habits of the heart: individualism and commitment in American life. Berkeley: University of California Press.

[R12] Blake-Beard S, Bayne ML, Crosby FJ, & Muller CB (2011). Matching by race and gender in mentoring relationships: Keeping our eyes on the prize. Journal of Social Issues, 67, 622–643. 10.1111/j.1540-4560.2011.01717.x.

[R13] Bonilla-Silva E (2003). Racism without racists: color-blind racism and the persistence of racial inequality in the United States. Lanham: Rowman & Littlefield.

[R14] Bronfenbrenner U (1979). The ecology of human development: experiments by nature and design. Cambridge: Harvard University Press.

[R15] Byars-Winston A, Womack VY, Butz AR, McGee R, Quinn SC, Utzerath E, (2018). Pilot study of an intervention to increase cultural awareness in research mentoring: implications for diversifying the scientific workforce. Journal of Clinical and Translational Science, 2, 86–94. 10.1017/cts.2018.25. 30338131 PMC6191051

[R16] Carpi A, Ronan DM, Falconer HM, & Lents NH (2017). Cultivating minority scientists: undergraduate research increases self-efficacy and career ambitions for underrepresented students in STEM. Journal of Research in Science Teaching, 54, 169–194. 10.1002/tea.21341.

[R17] Crenshaw K, Gotanda N, Peller G, & Thomas K (Eds.). (1995). Critical race theory: the key writings that formed the movement. New York: The New Press.

[R18] DiAngelo R (2011). White fragility. International Journal of Critical Pedagogy, 3, 54–70.

[R19] DiAngelo R (2018). White fragility: why it’s so hard for white people to talk about racism. Boston: Beacon Press.

[R20] Dodson JE, Montgomery BL, & Brown LJ (2009). “Taking the fifth”: mentoring students whose cultural communities were not historically structured into U.S. higher education. Innovative Higher Education, 34, 185–199. 10.1007/s10755-009-9099-y.

[R21] Fabrigar LR, MacDonald TK, & Wegener DT (2005). The structure of attitudes. In Albarracín D, Johnson BT, & Zanna MP (Eds.), The handbook of attitudes (pp. 79–124). Mahwah: Lawrence Erlbaum Associates.

[R22] Freire P (1970). Pedagogy of the oppressed. New York: Herder and Herder.

[R23] Gaertner SL, & Dovidio JF (1986). The aversive form of racism. In Dovidio JF & Gaertner SL (Eds.), Prejudice, discrimination, and racism (pp. 61–89). San Diego: Academic Press.

[R24] Gibbons A (1993). White men can mentor: help from the majority. Science, 262, 1130–1134. 10.1126/science.262.5136.1130. 17782070

[R25] Giroux HA (2003). Spectacles of race and pedagogies of denial: anti-black racist pedagogy under the reign of neoliberalism. Communication Education, 52, 191–211. 10.1080/0363452032000156190.

[R26] Graves II AT (2013). Mentee vs. protégé: what’s in a name? Retrieved from http://andersontgraves.blogspot.com/2013/04/mentee-vs-protege-whats-in-name.html.

[R27] Harber KD, Stafford R, & Kennedy KA (2010). The positive feedback bias as a response to self-image threat. British Journal of Social Psychology, 49, 207–218. 10.1348/014466609X473956. 19843351

[R28] Harris CI (1993). Whiteness as property. Harvard Law Review, 106, 1707–1791. Retrieved from https://papers.ssrn.com/sol3/papers.cfm?abstract_id=927850.

[R29] Hassouneh-Phillips D, & Beckett A (2003). An education in racism. Journal of Nursing Education, 42, 258–265. 10.3928/0148-4834-20030601-08. 12814216

[R30] Hendrix KG (1998). Student perceptions of the influence of race on professor credibility. Journal of Black Studies, 28, 738–763. 10.1177/002193479802800604.

[R31] Henry PJ, & Sears DO (2008). Symbolic and modern racism. In Moore JH (Ed.), Encyclopedia of race and racism (Vol. 3, pp. 111–112). Farmington Hills: Macmillan Reference.

[R32] Holoien DS, & Shelton JN (2012). You deplete me: the cognitive costs of colorblindness on ethnic minorities. Journal of Experimental Social Psychology, 48, 562–565. 10.1016/j.jesp.2011.09.010.

[R33] hooks B (1995). Killing rage: ending racism. New York: Henry Holt & Company.

[R34] Hrabowski III FA (2012). Broadening participation in the American STEM workforce. BioScience, 62, 325–326. 10.1525/bio.2012.62.4.2.

[R35] Ignatiev N (1995). How the Irish became white. New York: Routledge.

[R36] Jackman MR (1994). The velvet glove: paternalism and conflict in gender, class, and race relations. Los Angeles: University of California Press.

[R37] Jackson AP, Smith SA, & Hill CL (2003). Academic persistence among Native American college students. Journal of College Student Development, 44, 548–565. 10.1353/csd.2003.0039.

[R38] Jacobs JA, & Winslow SE (2004). Overworked faculty: job stresses and family demands. ANNALS of the American Academy of Political and Social Science, 596, 104–129. 10.1177/0002716204268185.

[R39] Jones CP (2002). Confronting institutionalized racism. Phylon, 50, 7–22. 10.2307/4149999.

[R40] Jost JT, & Banaji MR (1994). The role of stereotyping in system-justification and the production of false consciousness. British Journal of Social Psychology, 33, 1–27. 10.1111/j.2044-8309.1994.tb01008.x.

[R41] Jost JT,& Burgess D (2000). Attitudinal ambivalence and the conflict between group and system justification motives in low status groups. Personality and Social Psychology Bulletin, 26, 293–305. 10.1177/0146167200265003.

[R42] Jost JT, & Hunyady O (2002). The psychology of system-justification and the palliative function of ideology. European Review of Social Psychology, 13, 111–153. 10.1080/10463280240000046.

[R43] Jost JT, Banaji MR, & Nosek BA (2004). A decade of system justification theory: accumulated evidence of conscious and unconscious bolstering of the status quo. Political Psychology, 25, 881–919. 10.31234/osf.io/6ue35.

[R44] Kagan S (1992). Cooperative learning. San Juan Capistrano: Resources for Teachers.

[R45] Kendi IX (2016). Stamped from the beginning: the definitive history of racist ideas in America. New York: Nation Books.

[R46] Kim YK, & Sax LJ (2009). Student–faculty interaction in research universities: differences by student gender, race, social class, and first-generation status. Research in Higher Education, 50, 437–459. 10.1007/s11162-009-9127-x.

[R47] King JE (1991). Dysconscious racism: Ideology, identity, and the miseducation of teachers. Journal of Negro Education, 60, 133–146. 10.2307/2295605.

[R48] Kosoko-Lasaki O, Sonnino RE, & Voytko ML (2006). Mentoring for women and underrepresented minority faculty and institutions of higher education. Journal of the National Medical Association, 98, 1449–1459. 17019912 PMC2569723

[R49] Kruglanski AW, & Stroebe W (2005). The influence of beliefs and goals on attitudes: issues of structure, function, and dynamics. In Albarracín D, Johnson BT, & Zanna MP (Eds.), The handbook of attitudes (pp. 323–368). Mahwah: Lawrence Erlbaum Associates.

[R50] Ladson-Billings G (1998). Just what is critical race theory and what’s it doing in a nice field like education? International Journal of Qualitative Studies in Education, 11, 7–24. 10.1080/095183998236863.

[R51] Ledesma MC, & Calderón D (2015). Critical race theory in education: a review of past literature and a look to the future. Qualitative Inquiry, 21, 206–222. 10.1177/1077800414557825.

[R52] Lewis-Charp H (2003). Breaking the silence: white students’ perspectives on race in multiracial schools. Phi Delta Kappan, 85, 279–285. 10.1177/003172170308500407.

[R53] Luna N, & Revilla AT (2013). Understanding Latina/o school pushout: experiences of students who left school before graduating. Journal of Latinos and Education, 12, 22–37. 10.1080/15348431.2012.734247.

[R54] Malik K (2000). Universalism and difference in discourses of race. Review of International Studies, 26, 155–177. 10.1017/s0260210500001558.

[R55] Martín-Baró I (1994). Writings for a liberation psychology. Cambridge: Harvard University Press.

[R56] Maxwell N (2007). From knowledge to wisdom: the need for an academic revolution. London Review of Education, 5, 97–115. 10.1080/14748460701440350.

[R57] McCoy DL, Winkle-Wagner R, & Luedke CL (2015). Colorblind mentoring? Exploring white faculty mentoring of students of color. Journal of Diversity in Higher Education, 8, 225–242. 10.1037/a0038676.

[R58] McGee R, Saran S, & Krulwich TA (2012). Diversity in the biomedical research workforce: developing talent. Mount Sinai Journal of Medicine: A Journal of Translational and Personalized Medicine, 79, 397–411. 10.1002/msj.21310. PMC3375909 22678863

[R59] [NIH WGDBRW] National Institutes of Health: Working Group on Diversity in the Biomedical Research Workforce. (2012). Draft report of the advisory committee to the director. Retrieved from http://acd.od.nih.gov/Diversity%20in%20the%20Biomedical%20Research%20Workforce%20Report.pdf

[R60] Ortiz L, & Jani J (2010). Criticalrace theory: a transformational model for teaching diversity. Journal of Social Work Education, 46, 175–193. 10.5175/jswe.2010.200900070.

[R61] Plunkett S, Saetermoe CL, & Quilici JL (2014). An evaluation of the undergraduate Career Opportunities in Research (COR) program. Council on Undergraduate Research Quarterly, 35, 36–42.

[R62] Ponjuan L (2011). Recruiting and retaining Latino faculty members: The missing piece to Latino student success. Thought & Action, 27, 99–110.

[R63] Potter SJ, Abrams E, Townson L, & Williams JE (2009). Mentoring undergraduate researchers: faculty mentors’ perceptions of the challenges and benefits of the research relationship. Journal of College Teaching & Learning, 6, 17–30. 10.19030/tlc.v6i6.1131.

[R64] Prunuske AJ, Wilson J, Walls M, & Clarke B (2013). Experiences of mentors training underrepresented undergraduates in the research laboratory. CBE—Life Sciences Education, 14, 403–409. 10.1187/cbe.13-02-0043. PMC3763008 24006389

[R65] Quaye SJ, Griffin KA, & Museus SD (2015). Engaging students of color. In Quaye SJ & Harper SR (Eds.), Student engagement in higher education: theoretical perspectives and practical approaches for diverse populations (2nd ed., pp. 15–36). New York: Routledge.

[R66] Russell JE, D’Costa AR, Runck C, Barnes DW, Barrera AL, Hurst-Kennedy J, (2015). Bridging the undergraduate curriculum using an integrated course-embedded undergraduate research experience (ICURE). CBE—Life Sciences Education, 14, 1–10. 10.1187/cbe.14-09-0151. PMC4353079 25681416

[R67] Sabat IE, Lindsey AP, King EB, Ahmad AS, Membere A, & Arena DF (2017). How prior knowledge of LGB identities alters the effects of workplace disclosure. Journal of Vocational Behavior, 103, 56–70. 10.1016/j.jvb.2017.09.001.

[R68] Saetermoe CL, Chavira G, Khachikian CS, Boyns D, & Cabello B (2017). Critical race theory as a bridge in science training: the California State University, Northridge BUILD PODER program. Biomedical Central Proceedings, 11, 41–55. 10.1186/s12919-017-0089-2. PMC5773894 29375662

[R69] Salter PS, & Haugen AD (2017). Critical race studies in psychology. In Gough B (Ed.), The Palgrave handbook of critical social psychology (pp. 123–145). London: Palgrave Macmillan. 10.1057/978-1-137-51018-1_7.

[R70] Santos SJ, & Reigadas ET (2004). Understanding the student-faculty mentoring process: its effects on at-risk university students. Journal of College Student Retention: Research, Theory & Practice, 6, 337–357. 10.2190/kgvc-7218-dper-rmbc.

[R71] Seymour E, & Hewitt NM (1997). Talking about leaving: why undergraduates leave the sciences. Boulder: Westview Press.

[R72] Shain BA (1994). The myth of American individualism: the Protestant origins of American political thought. Princeton: Princeton University Press.

[R73] Solomon RP, Portelli JP, Daniel B-J, & Campbell A (2005). The discourse of denial: how white teacher candidates construct race, racism, and ‘white privilege’. Race Ethnicity and Education, 8, 147–169. 10.1080/13613320500110519.

[R74] Solórzano DG, & Yosso TJ (2002). Critical race methodology: counter-storytelling as an analytical framework for education research. Qualitative Inquiry, 8, 23–44. 10.1177/1077800402008001003.

[R75] Suarez-Balcazar Y, Orellana-Damacela L, Portillo N, Rowan JM, & Andrews-Guillen C (2003). Experiences of differential treatment among college students of color. Journal of Higher Education, 74, 428–444. 10.1353/jhe.2003.0026.

[R76] Sue DW, Torino GC, Capodilupo CM, Rivera DP, & Lin AI (2009). How white faculty perceive and react to difficult dialogues on race. The Counseling Psychologist, 37, 1090–1115. 10.1177/0011000009340443.

[R77] Tatum BD (1997). “Why are all the black kids sitting together in the cafeteria?”: and other conversations about race. New York: BasicBooks. 10.1126/science.abn0865 34822268

[R78] Thomas DA, & Ely RJ (1996). Making differences matter: a new paradigm for managing diversity. Harvard Business Review, 74, 79–90.

[R79] [U.S. DOEd NCES] United States Department of Education: National Center for Education Statistics. (2018). The condition of education 2018. Washington, DC: National Center for Education Statistics. Retrieved from https://nces.ed.gov/pubs2018/2018144.pdf.

[R80] Vargas JH, & Kemmelmeier M (2012). Ethnicity and contemporary American culture: a meta-analytic investigation of horizontal–vertical individualism–collectivism. Journal of Cross-Cultural Psychology, 44, 195–222. 10.1177/0022022112443733.

[R81] Viesca KM, Torres AS, Barnatt J, & Piazza P (2013). When claiming to teach for social justice is not enough: majoritarian stories of race, difference, and meritocracy. Berkeley Review of Education, 4, 97–122. 10.5070/b84110002.

[R82] Wallace T, & Brand BR (2012). Using critical race theory to analyze science teachers’ culturally responsive practices. Cultural Studies of Science Education, 7, 341–374. 10.1007/s11422-012-9380-8.

[R83] Weinberg SL (2008). Monitoring faculty diversity: the need for a more granular approach. Journal of Higher Education, 79, 365–387. 10.1353/jhe.0.0014.

[R84] Whitley BE Jr., & Kite ME (2010). The psychology of prejudice and discrimination (2nd ed.). Belmont: Thomson-Wadsworth.

[R85] Wingfield AH (2015). Color-blindness is counterproductive: many sociologists argue that ideologies claiming not to see race risk ignoring discrimination. Retrieved from https://www.theatlantic.com/politics/archive/2015/09/color-blindness-is-counterproductive/405037

[R86] Wise T (2017). THE GREAT WHITE HOAX: Donald Trump & the politics of race & class in America. Northampton: Media Education Foundation.

[R87] Yosso TJ (2002). Toward a critical race theory curriculum. Equity & Excellence in Education, 35, 93–107. 10.1080/713845283.

[R88] Yosso TJ (2005). Whose culture has capital? A critical race theory discussion of community cultural wealth. Race Ethnicity and Education, 8, 69–91. 10.1080/1361332052000341006.

[R89] Young G, & Davis-Russell E (2002). The vicissitudes of cultural competence: dealing with difficult classroom dialogue. In Davis-Russell E (Ed.), The California School of Professional Psychology handbook of multicultural education, research, intervention, and training (pp. 37–53). San Francisco: Jossey-Bass.

